# Supplementary material for: Early-Life Supplementation of Bovine Milk Osteopontin Supports Neurodevelopment and Influences Exploratory Behavior
Source: Nutrients. 2020 Jul 24;12(8):2206. doi: 10.3390/nu12082206 (PMC7469054; doi:10.3390/nu12082206)
Supplement: Supplementary file 1 [file nutrients-12-02206-s001.pdf]

Supplemental Table S1. Absolute brain volumes (mm^3)[1]

Region of Interest	Diet		Pooled	
	Control	Test	SEM	P-value
Whole brain	58,731.55	57,175.19	1,724.208	0.332
Grey matter	36,987.00	38,380.00	4,188.730	0.680
White matter	14,949.00	14,912.00	720.690	0.958
Cerebrospinal fluid	4,345.00	3,877.63	1954.770	0.807
Cerebellum	5,950.67	5,753.80	175.521	0.217
Cerebral aqueduct	14.15	14.12	0.426	0.953
Corpus callosum	227.70	232.85	7.607	0.516
Fourth ventricle	17.77	16.89	0.814	0.442
Hypothalamus	85.79	84.82	2.596	0.688
Lateral ventricle	318.06	326.79	10.938	0.447
Left caudate	202.82	206.80	5.526	0.603
Left cortex	15,283.54	15,473.25	341.629	0.692
Left hippocampus	273.22	275.00	5.483	0.812
Left inferior colliculi	66.21	64.37	1.533	0.395
Left internal capsule	475.69	489.49	10.613	0.359
Left olfactory bulb	1,092.57	1,130.78	37.270	0.478
Left putamen-globus pallidus	112.44	116.39	2.673	0.299
Left superior colliculi	160.82	156.18	2.437	0.138
Medulla	1,417.80	1,397.01	37.308	0.678
Midbrain	1,982.71	1,935.40	31.066	0.279
Pons	1,193.80	1,164.04	30.049	0.483
Right caudate	211.88	217.71	10.438	0.495
Right cortex	15,222.65	15,376.15	449.515	0.717
Right hippocampus	284.84	294.81	8.860	0.345
Right inferior colliculi	67.29	65.60	1.421	0.400
Right internal capsule	460.57	480.61	17.214	0.151
Right olfactory bulb	1,025.94	1,117.49	57.566	0.223
Right putamen-globus pallidus	106.63	109.96	4.271	0.279
Right superior colliculi	166.02	163.80	3.901	0.592
Thalamus	1,070.19	1,068.49	30.356	0.948
Third ventricle	19.45	19.20	0.676	0.798

[1]Pigs received diets containing 0 (Control) or 250 (Test) mg of bovine milk OPN per L of mixed milk replacer during a 30-d feeding study (n = 11 male pigs for control group and 10 male pigs for test group). Abbreviation: OPN, osteopontin; SEM, standard error of mean.

Supplemental Table S2. Axial diffusivity (AD; × $10^{-3}/mm^2/s$)[1]

Region of Interest	Diet		Pooled	
	Control	Test	SEM	P-value
Corpus callosum	0.86	0.83	0.010	0.086
Cerebellum	0.65	0.62	0.010	0.119
Left caudate	0.88	0.85	0.024	0.217
Left hippocampus	0.88	0.86	0.036	0.283
Left internal capsule	0.83	0.83	0.018	0.883
Right caudate	0.85	0.86	0.017	0.785
Right hippocampus	0.87	0.85	0.017	0.524
Right internal capsule	0.84	0.82	0.012	0.225
Left side	0.77	0.76	0.006	0.169
Right side	0.76	0.76	0.007	0.966
Thalamus	0.76	0.75	0.007	0.125
T1 white matter	0.77	0.77	0.006	0.458
Average AD mask	0.74	0.74	0.011	0.579

[1]Pigs received diets containing 0 (Control) or 250 (Test) mg of bovine milk OPN per L of mixed milk replacer during a 30-d feeding study (n = 11 male pigs for control group and 10 male pigs for test group). Data presented are least square means and P-values from mixed model ANOVA. Abbreviation: OPN, osteopontin; SEM, standard error of mean.

Supplemental Table S3. Mean diffusivity (MD; × $10^{-3}/mm^2/s$)[1]

Region of Interest	Diet		Pooled	
	Control	Test	SEM	P-value
Corpus callosum	0.62	0.63	0.025	0.126
Cerebellum	0.51	0.50	0.006	0.245
Left caudate	0.65	0.64	0.023	0.233
Left hippocampus	0.63	0.62	0.016	0.341
Left internal capsule	0.49	0.48	0.009	0.441
Right caudate	0.65	0.65	0.011	0.863
Right hippocampus	0.62	0.61	0.012	0.674
Right internal capsule	0.49	0.49	0.010	0.862
Left side	0.55	0.54	0.004	0.132
Right side	0.54	0.54	0.005	0.904
Thalamus	0.58	0.57	0.005	0.068
T1 white matter	0.55	0.54	0.004	0.407
Average MD mask	0.55	0.55	0.008	0.538

[1]Pigs received diets containing 0 (Control) or 250 (Test) mg of bovine milk OPN per L of mixed milk replacer during a 30-d feeding study (n = 11 male pigs for control group and 10 male pigs for test group). Data presented are least square means and P-values from mixed model ANOVA. Abbreviation: OPN, osteopontin; SEM, standard error of mean.

Supplemental Table S4. Radial diffusivity (RD; × $10^{-3}/mm^2/s$)[1]

Region of Interest	Diet		Pooled	
	**Control**	**Test**	**SEM**	**P-Value**
Corpus callosum	0.50	0.53	0.038	**0.008**
Cerebellum	0.44	0.44	0.005	0.293
Left caudate	0.54	0.53	0.024	0.479
Left hippocampus	0.51	0.50	0.010	0.552
Left internal capsule	0.32	0.32	0.014	0.797
Right caudate	0.55	0.55	0.011	0.946
Right hippocampus	0.51	0.49	0.008	0.236
Right internal capsule	0.32	0.32	0.017	0.818
Left side	0.44	0.43	0.004	0.128
Right side	0.43	0.43	0.003	0.250
Thalamus	0.48	0.48	0.006	0.323
T1 white matter	0.43	0.43	0.004	0.430
Average RD mask	0.45	0.45	0.007	0.503

[1]Pigs received diets containing 0 (Control) or 250 (Test) mg of bovine milk OPN per L of mixed milk replacer during a 30-d feeding study (n = 11 male pigs for control group and 10 male pigs for test group). Data presented are least square means and P-values from mixed model ANOVA. Abbreviation: OPN, osteopontin; SEM, standard error of mean.
